# COX Inhibitory and Cytotoxic Naphthoketal-Bearing Polyketides from *Sparticola junci*

**DOI:** 10.3390/ijms222212379

**Published:** 2021-11-17

**Authors:** Katherine Yasmin M. Garcia, Mark Tristan J. Quimque, Gian Primahana, Andreas Ratzenböck, Mark Joseph B. Cano, Jeremiah Francis A. Llaguno, Hans-Martin Dahse, Chayanard Phukhamsakda, Frank Surup, Marc Stadler, Allan Patrick G. Macabeo

**Affiliations:** 1 The Graduate School, University of Santo Tomas, España Blvd., Manila 1015, Philippines; katherineyasmin.garcia.gs@ust.edu.ph (K.Y.M.G.); marktristan.quimque@g.msuiit.edu.ph (M.T.J.Q.); markjoseph.cano.gs@ust.edu.ph (M.J.B.C.); 2 Laboratory for Organic Reactivity, Discovery and Synthesis (LORDS), Research Center for the Natural and Applied Sciences, University of Santo Tomas, España Blvd., Manila 1015, Philippines; jeremiahfrancis.llaguno.sci@ust.edu.ph; 3 Chemistry Department, College of Science and Mathematics, Mindanao State University Iligan Institute of Technology, Tibanga, Iligan City 9200, Philippines; 4 Department of Microbial Drugs, Helmholtz Centre for Infection Research and German Centre for Infection Research (DZIF), Partner Site Hannover/Braunschweig, Inhoffenstrasse 7, 38124 Braunschweig, Germany; gian.primahana@helmholtz-hzi.de (G.P.); frank.surup@helmholtz-hzi.de (F.S.); marc.stadler@helmholtz-hzi.de (M.S.); 5 Institute of Microbiology, Technische Universität Braunschweig, Spielmannstraße 7, 38106 Braunschweig, Germany; 6 Research Center for Chemistry, National Research and Innovation Agency (BRIN), Kawasan Puspitek, Serpong, Tangerang Selatan 15314, Indonesia; 7 Institut für Organische Chemie, Universität Regensburg, Universitätstrasse 31, 93053 Regensburg, Germany; andreas.ratzenboeck@chemie.uni-regensburg.de; 8 Leibniz-Institute for Natural Product Research and Infection Biology, Hans-Knöll-Institute (HKI), 07745 Jena, Germany; hans-martin.dahse@hki-jena.de; 9 Center of Excellence in Fungal Research, Mae Fah Luang University, Chiang Rai 57100, Thailand; chayanard91@gmail.com; 10 Institute of Plant Protection, College of Agriculture, Jilin Agricultural University, Changchun 130118, China

**Keywords:** *Sparticola junci*, structure elucidation, ECD-TDDFT, COX inhibitory, molecular docking, antiproliferative, cytotoxic

## Abstract

Axenic fermentation on solid rice of the saprobic fungus *Sparticola junci* afforded two new highly oxidized naphthalenoid polyketide derivatives, sparticatechol A (**1**) and sparticolin H (**2**) along with sparticolin A (**3**). The structures of **1** and **2** were elucidated on the basis of their NMR and HR-ESIMS spectroscopic data. Assignment of absolute configurations was performed using electronic circular dichroism (ECD) experiments and Time-Dependent Density Functional Theory (TDDFT) calculations. Compounds **1–3** were evaluated for COX inhibitory, antiproliferative, cytotoxic and antimicrobial activities. Compounds **1** and **2** exhibited strong inhibitory activities against COX-1 and COX-2. Molecular docking analysis of **1** conferred favorable binding against COX-2. Sparticolin H (**2**) and A (**3**) showed a moderate antiproliferative effect against myelogenous leukemia K-562 cells and weak cytotoxicity against HeLa and mouse fibroblast cells.

## 1. Introduction

Plant-associated fungi constitutes a myriad and relatively less explored repository of biologically active natural products that may serve as key starting points for pharmaceutical drug development, biotechnology and agrochemical applications [1,2,3]. The Dothideomycetes, which comprise the largest taxa in the Ascomycota, are known to produce fungal secondary metabolites, possessing distinct chemical structures associated with biological activities that have gained considerable interest. Several genera of Dothideomycetes elaborate oxidized bisnaphthalenoids that consist of 1,8-dihydroxynaphthalene-derived units and a decalin moiety bridged through spiroketal linkages [4]. Since the first discovery of the antibacterial diketo-bisnaphthalenoid natural product MK 3018 isolated from the fungal culture of *Tetraploa aristata* [5], a growing number of naphthospiroketal derivatives have been reported, originating from a diverse group of fungi and considered to be potential drug leads exhibiting a wide range of biological properties [6,7,8,9,10,11]. MK 3018 exhibited potent in vitro cytotoxicity against P388 murine leukemia cell line [6].

The 1,8-dihydroxynaphthalene polyketide *spiro*-mamakone is the first example of *nor*-spirodioxynaphthalene derivative featuring a *spiro*-nonaphthadiene skeleton. Its isolation spurred biosynthetic studies which have illustrated oxidative coupling and rearrangement of naphthalene subunits followed by decarboxylation and ring closure in the biogenetic pathways of *nor-* and *nor-seco* derivatives [12]. Our initial efforts in exploring the secondary metabolites of *Sparticola junci* (family Sporormiaceae), an ascomycetous saprophytic species previously isolated from the decomposing branches of the Spanish broom, *Spartium junceum* (family Fabaceae), has led to the isolation of additional antimicrobial and cytotoxic *nor-seco* congeners. These biologically active spirodioxynaphthalene derivatives showcase unprecedented structural frameworks that bear carboxyalkylidene–cyclopentanoid, carboxyl-functionalized oxabicyclo [3.3.0]octane, annelated 2-cyclopentenone/δ-lactone, and precursor catechol-bearing sub-structures [13,14].

In our continuing search of biologically active secondary metabolites from Dothideomycetes fungi [13,14,15,16], we herein report the isolation and structure identification of two new spirodioxynaphthalene derivatives, hitherto referred to as sparticatechol A (**1**) and sparticolin H (**2**) along with the known polyketide, sparticolin A (**3**) from the fermented solid rice culture and evaluation of their cyclooxygenase inhibitory, antiproliferative, cytotoxic and antimicrobial activities. To complement the observed biological studies, in silico molecular docking simulations on cyclooxygenases, and determination of drug-likeness, adsorption, distribution, metabolism, excretion, and toxicity (ADME-Tox) of the bioactive naphthoketal derivatives **1**–**3** were also carried out. 

## 2. Results and Discussion

**Figure 1:**
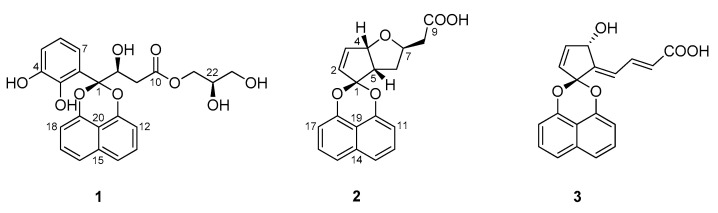
Dioxynaphthalenoids **1–3** from *Sparticola junci*.

The ethyl acetate (EtOAc) extract of *Sparticola junci* obtained after fermentation on a solid rice medium was partitioned between *n*-heptane and 10% aqueous MeOH. The resulting aqueous methanolic crude extract was purified using gradient elution vacuum liquid chromatography followed by semi-preparative HPLC to afford metabolites **1–3** (Figure 1). This study highlights the structure elucidation and biological activity determination of new naphthoketals **1** and **2** based on NMR spectroscopic data, HR-ESIMS and ECD-TDDFT calculations. The known *nor-seco* spirodioxynapthalenoid, sparticolin A (**3**) was identified by comparing its physicochemical and NMR spectroscopic data with those reported in the literature. Compound **3** was previously obtained from the submerged fermentation culture of *S. junci* and exhibited weak antimicrobial and cytotoxic activities.

Sparticatechol A (**1**) was obtained as an optically active reddish-brown syrup. The molecular formula C_23_H_22_O_9_, indicating 13 degrees of unsaturation, was established based on the protonated molecular ion peak at *m/z* 443.1330 [M + H]^+^ of its positive-ion HRESIMS. This is consistent with the number of proton and carbon peaks detected in its ^1^H and ^13^C NMR spectroscopic data (Table 1). Detailed examination of the ^1^H, ^13^C, and HSQC-DEPT NMR spectroscopic data revealed the presence of a carboxylic acid carbon, a ketal carbon, seven non-protonated aromatic carbons (four oxygenated and three non-oxygenated), nine aromatic methines, three *sp*^3^ methylene and two oxygenated *sp*^3^ methine carbon.

**Figure 2:**
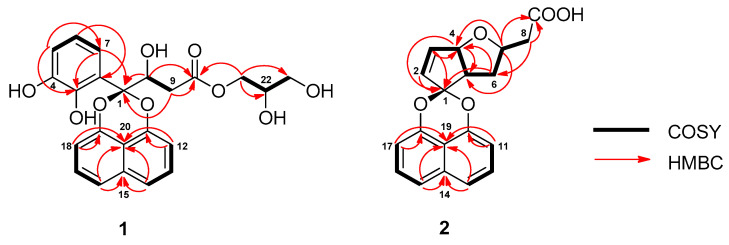
COSY and HMBC correlations in **1** and **2**.

Analysis of the COSY spectrum identified two isolated three distinct spin systems of *δ_H_* 7.37 (2H, *dd*, *J* = 8.3 Hz, H-13/H-17) and *δ_H_* 7.02 (2H, *dd*, *J* = 8.3, 1.3 Hz, H-12/H-18), and of *δ_H_* 7.39 (2H, *dd*, *J* = 8.3, 1.3 Hz, H-14/H-16) corresponding to C-12 to C-14 and to C-16 to C-18 naphthalene subunits of **1**. These aromatic protons are mutually *ortho*-coupled to each other (*J* = 8.3 Hz). HMBC correlations of H-14/H-16 with two non-protonated *sp^2^* carbons *δ_C_* 135.5 (C-15) and *δ_C_* 115.5 (C-20), along with long range correlations of H-12 and H-13 with *δ_C_* 148.7 (C-11), and H-17 and H-18 with *δ_C_* 148.9 (C-19), suggested that subunits C-12 to C-14 and C-16 to C-18 are linked to C-15 and C-20, leading to the construction of a naphthalene fragment. The midfield chemical shifts observed with C-11 and C-19 proposed 1,8-dioxygenation in the naphthalene unit. The remaining portion of **1** was constructed through analysis of COSY and HSQC-DEPT spectroscopic data, revealing aromatic signals representative of a 1,2,3-trisubstituted benzene ring at *δ_H_* 6.59 (1H, *dd*, *J* = 7.9, 1.5 Hz, H-5), *δ_H_* 6.42 (1H, *t*, *J* = 7.9 Hz, H-6), *δ_H_* 6.80 (1H, *dd*, *J* = 7.9, 1.5 Hz, H-7), a two-proton spin system consisting of the oxygenated *sp*^3^ methine *δ_H_* 4.81 (1H, *dd*, *J* = 14.0, 3.1, H-8) in the *β*-carbon coupled to the diastereotopic methylene protons at *δ_H_* 2.68 (1H, *dd*, *J* = 15.6, 9.4 Hz, H-9a), 2.97 (1H, *dd*, *J* = 15.7, 3.2 Hz, H-9b) connected to the *β*-carbon, and a three-proton spin system at *δ_H_* 4.10 (1H, *td*, *J* = 11.4, 6.3 Hz, H-21a), 4.19 (1H, *td*, *J* = 11.0, 4.3 Hz, H-21b), *δ_H_* 3.80 (2H, *dd*, *J* = 18.3, 10.4 Hz, H-22*)*, *δ_H_* 3.56 (2H, *dt*, *J* = 5.7, 4.8, 2.3 Hz, H-23) (Figure 2) arising from the glycerol subunit. HMBC correlations of *δ_H_* 6.59 (H-5) with *δ_C_* 145.1 (C-3) and *δ_C_* 147.3 (C-4) and of *δ_H_* 6.80 (H-7) with *δ_C_* 105.1 (C-1) and C-3 allowed the identification of a substituted catechol fragment. In addition, the correlations of *δ_H_* 4.10, 4.19 (H_2_-21) with an oxygenated *sp*^3^ methine *δ_C_* 71.1 (C-22) and an oxygenated *sp*^3^ methylene *δ_C_* 64.0 (C-23) suggested the presence of dihydroxypropyl residue. Further HMBC correlations of *δ_H_* 4.10, 4.19 (H_2_-21), *δ_H_* 4.81 (H-8) and *δ_H_* 2.68, 2.97 (H_2_-9) with the carboxylic carbon *δ_C_* 173.1 (C-10) established the connectivities of the glycerol residue and the α-hydroxy carboxylic acid fragment, allowing for the construction of a glyceryl-*β*-hydroxybutanoate motif. Finally, HMBC correlations of H-7, H-8, and H_2_-9 with the *spiro*-ketal carbon *δ_C_* 105.1 (C-1) led to the attachment of the catechol moiety and the glyceryl-*β*-hydroxybutanoate motif to the 1,8-dioxygenated naphthalene subunit. On the basis of the data discussed above, the planar structure of **1** was identified as 2,3-dihydroxypropyl-3-(2-(2,3-dihydroxyphenyl)naphtho[1,8-de][1,3]dioxin-2-yl)-3-hydroxypropanoate.

**Figure 3:**
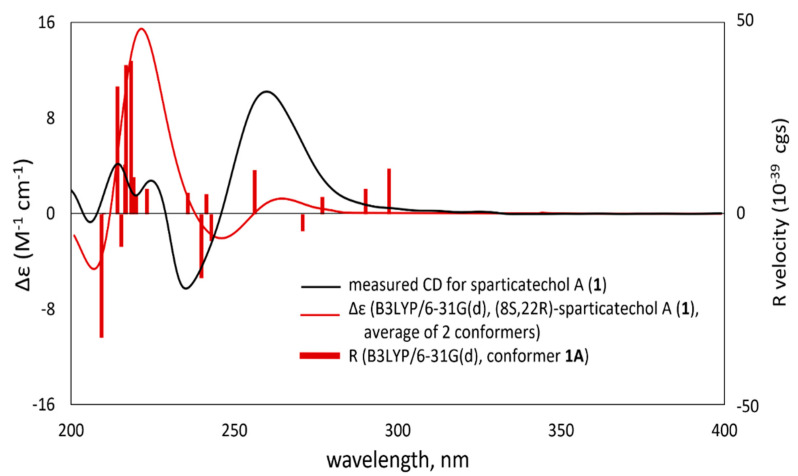
Experimental ECD spectrum of sparticatechol A (**1**, black solid curve) compared with B3LYP/6-31G(d)-calculated ECD spectra (red solid curve) for the B3LYP/6-31G(d)-optimized conformers of (8*S*,22*R*)-**1**.

TDDFT-ECD calculations were performed to determine the absolute configuration of sparticatechol A (**1**) unambiguously. Prior to ECD calculations, a conformational analysis was done using an MMFF94 force field to search for conformers with room-temperature equilibrium population > 1%. Geometry re-optimization via DFT calculations at a B3LYP/6-31G(d) basis set afforded two stable conformers with a configurational assignment arbitrarily chosen as (8*S*,22*R*) based on the previously determined configuration of the catechol-bearing naphthoketal [13] and the naturally occurring (2*R*)-glyceraldehyde-3-phosphate [17,18]. The two stable conformers differed in the orientation, mainly due to the flexibility of the ester chain, particularly along the C8-C9 and C22-C23 axes. Conformer 1A (95.03%) adapted almost an anti-staggered conformation with respect to the C7 *spiro*-ketal carbon and the C10 carbonyl carbon about the C8-C9 axis with a C7-C8-C9-C10 dihedral angle of 170.35°. Conformer 1B (4.97%), on the other hand, adapted a gauche configuration about the said axis; however, this was compensated by strong intramolecular hydrogen bonding between two hydroxy groups, C6(OH) and C22(OH). Moreover, along the C22-C23 axis, conformer 1B adapted an anti-staggered conformation. The 1A and 1B conformations were also supported by the ROESY correlations between H-8/H-7, H-8/H-9b, H-8/H-18, and H-6/H-22, respectively. Based on the harmonic vibrational frequency calculations, these conformers are confirmed stable. TDDFT calculations were carried out on both conformers at the following levels of theory/basis sets: B3LYP/6-31G(d), WB97XD/DGDZVP, and B97D/TZVP in the gas phase and polarizable continuum model (PCM) for acetonitrile. The experimental ECD spectrum of compound **1** showed two negative cotton effects (CE’s)–one at 206 nm and a more prominent peak at 246 nm. In the latter CE peak, the calculated ECD spectrum at B3LYP/6-31G(d) in gas phase exhibited a blue shift of about 10 nm. The use of other levels of theory such as WB97XD and B97D only increased the said blue shift. Overall, the Boltzmann-averaged ECD spectrum of sparticatechol A (Figure 3) calculated at B3LYP/6-31G(d) provided good agreement with the experimental ECD spectrum. Thus, the absolute configuration of sparticatechol A (**1**) is (8*S*,22*R*).

Sparticolin H (**2**) was isolated as a dark brown syrup. The HRESIMS of **2** showed a sodiated molecular ion peak at *m/z* 347.0889, corresponding to the molecular formula C_19_H_16_NaO_5_, indicating an index of hydrogen deficiency of 12. In addition to signals resonating for a 1,8-dioxynaphthalene subunit, detailed investigation of the ^1^H, ^13^C, (Table 1) and HSQC-DEPT NMR spectroscopic data revealed the presence of additional signals corresponding to a carboxylic acid, a ketal carbon, two *sp*^3^ methylene carbons, two olefinic methines, and three *sp*^3^ methines (two oxygenated and one non-oxygenated). The ^1^H-^1^H COSY NMR showed the presence of a seven-proton system corresponding to the C-2 to C-8 cyclopentanoid units in (Figure 2). Key HMBC correlations of *δ_H_* 5.88 (H-2), *δ_H_* 4.81 (H-3), *δ_H_* 5.07 (H-4), *δ_H_* 3.14 (H-5) with the *spiro*-ketal carbon *δ_C_* 111.0 (C-1) allowed the identification of C-4/C-5 disubstituted 2-cyclopentene motif connected to the dioxygenated naphthalene fragment. Further HMBC correlations of H-4 with *δ_C_* 78.7 (C-7) and H-7 with *δ_C_* 51.1 (C-5) suggested the annelation of a tetrahydrofuran moiety in the cyclopentene ring, establishing a 1-oxabicyclo[3.3.0]octane subunit. Finally, the HMBC correlations of H-7 and H-8 to the carboxylic carbon *δ_C_* 173.5 (C-9) allowed the determination of the gross structure of **2**. The vicinal coupling constant of 7 Hz (^3^*J*_H-4,H-5_) between H-4 and H-5 suggested a *cis* geometry. These NMR data correspond to a similar structural framework observed for sparticolin E, as described in our previous study [12]. In the NOESY spectrum, however, an NOE effect was only observed between H-4 and H-5. This analysis suggested that H-7 is spatially different from the two protons rendering an assignment of relative configurations of the chiral carbons as 4S*, 5R*, and 7R*. Thus, the structure of compound **2** was deduced as the C-7 epimer of sparticolin E.

**Figure 4:**
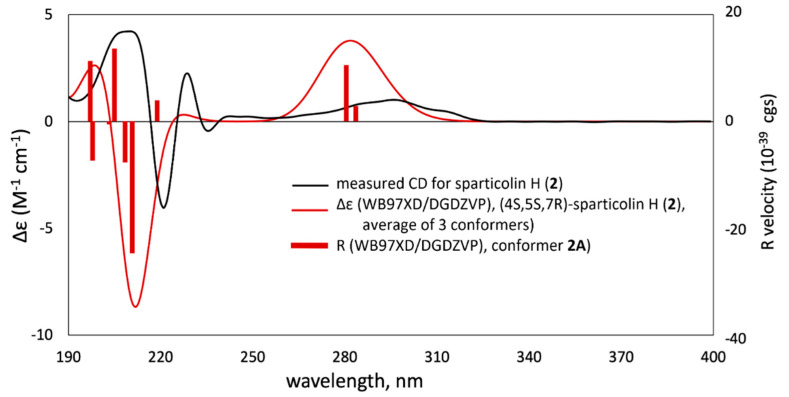
Experimental ECD spectrum of sparticolin H (**2**, black solid curve) compared with WB97XD/DGDZVP (PCM/MeCN)-calculated ECD spectra (red solid curve) for the WB97XD/DGDZVP-optimized conformers of (4*S*,5*S*,7*R*)-**2**.

Sparticolin H (**2**) was also subjected to TDDFT-ECD calculations to confirm its absolute configuration. Due to its rigid structure, conformational analysis of **2** yielded only one prevalent conformer with an equilibrium population of 99.62% with a pre-assigned (4*S*,5*S*,7*R*) configuration with two other low-energy conformers. All conformers are confirmed stable per harmonic vibrational frequency calculations. The theoretically obtained Boltzmann-averaged ECD spectrum of **2** at WB97XD/DGDZVP (PCM/MeCN) showed a good correlation with the experimental data (Figure 4), which strongly suggests that the absolute configuration of sparticolin H is (4*S*,5*S*,7*R*).

Compounds **1**–**3** were also evaluated for their cytotoxic activity against mouse fibroblasts L-929 (ACC 2), human cell lines like HeLa cells (ACC 57), KB-3-1 (ACC 158), squamous carcinoma A-431 (ACC 91), lung carcinoma A-549 (ACC 107), ovarian carcinoma SK-OV-3 (HTB-77), prostate cancer PC-3 (ACC 465), and breast adenocarcinoma MCF-7 (ACC 115). Among the isolated compounds, sparticolins H (**2**) and A (**3**) exhibited a moderate antiproliferative effect against myelogenous leukemia (Table 2). In addition, compound **2** displayed moderate cytotoxic properties against HeLa cervical cancer cell line and mouse fibroblast cell line L-929. All compounds were weakly inhibitory against *Candida albicans* (DSM 1665), *Mucor hiemalis* (DSM 2656), *Pichia anomala* (DSM 6766), *Rhodoturula glutinis* (DSM 10134), *Schizosaccharomyces pombe* (DSM 70572), *Bacillus subtilis* (DSM 10), *Chromobacterium violaceum* (DSM 30191), *Escherichia coli* (DSM 1116), *Acinetobacter baumannii* (DSM 30008), *Mycobacterium smegmatis* (ATCC 700084), *Pseudomonas aeruginosa* (DSM PA14), *Staphylococcus aureus* (DSM 346) and *Mycobacterium tuberculosis* H_37_Rv.

**Figure 5:**
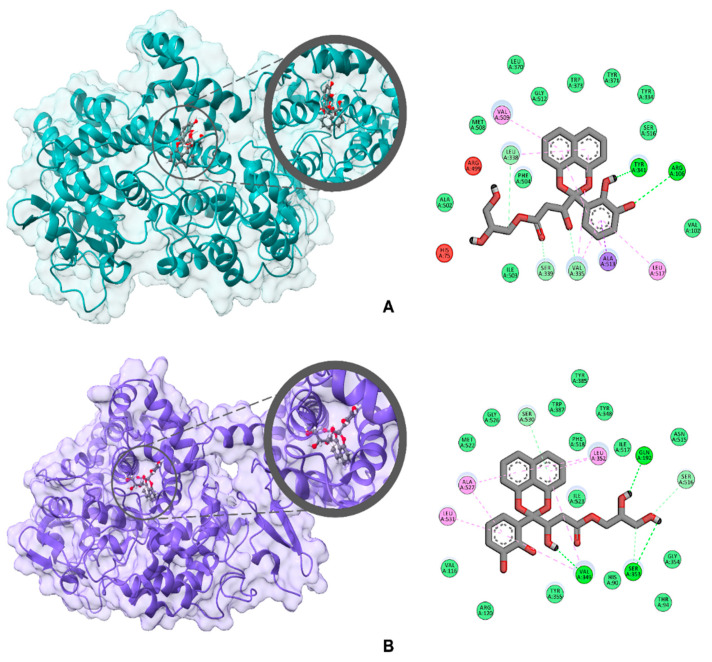
3D and 2D docked poses of sparticatechol A (1) against (**A**) COX-2 (PDB ID: 3LN1) and (**B**) COX-1 (PDB ID: 3KK6).

The anti-inflammatory properties of **1–3** were assessed in vitro via their inhibitory activities against COX-1 and COX-2 (Table 3). All compounds were found to have high COX-2 and COX-1 inhibitory activities, with compound **1** exhibiting the highest activity. The interesting COX inhibitory activity of **1** prompted us to investigate further its binding behavior, particularly the protein-ligand interactions, against COX-2 and COX-1 via in silico molecular docking analysis (Figure 5). All calculations were done on AutoDock Vina with COX-2 (PDB ID: 3LN1) and COX-1 (PDB ID: 3KK6) as receptors. Validation of the docking protocol was done through redocking of the native ligand Celecoxib at the conserved active site for non-steroidal anti-inflammatory drugs (NSAID), which resulted in RMSD values of 0.448 Å for COX-2 and 0.668 Å for COX-1; both RMSDs are less than 2 Å, which indicates that the computed ligand–protein conformations are good [19,20,21]. Sparticatechol A (**1**) exhibited a high binding affinity of −9.2 kcal/mol against COX-2. Attempts to direct the docking of the compound to other plausible binding sites of COX-2, such as the hydrophilic side chain and the POX region, provided weaker binding, and thus reveals a preferential binding of the compound to the NSAID active site. At the active site, compound **1** is stabilized through various intermolecular forces at different regions of the molecule. The dihydroxyphenyl fragment exhibited two conventional H-bonding against Arg106 and Tyr341, while the aromatic ring interacted with the following residues: Ala 513 via pi-sigma and Leu517 via pi-alkyl. The naphthoketal moiety, on the other hand, is attached via pi-alkyl interplay with Val335, Leu338, and Val509. Four carbon–hydrogen bond interactions (against Gln178, Val335, Leu338, and Ser339) can also be observed for the β-hydroxyester chain. Meanwhile, sparticatechol A (**1**) is bound to the active site of COX-1 with a binding energy of -7.5 kcal/mol. The naphthalene fragment of **1** displayed two prominent pi interactions, particularly pi-donor hydrogen bonds with Ser530 and pi-alkyl attraction with Leu352 and Val 349. Several aromatic pi-alkyl interactions can also be observed between the dihydroxyphenyl fragment against Val349, Ala527, and Leu531. Moreover, three conventional hydrogen bonds helped strengthen **1**’s attachment to the enzyme, specifically between the following: C8(OH) and Val349, C22(OH) and Gln192, and C23(OH) and Ser353.

**Figure 6:**
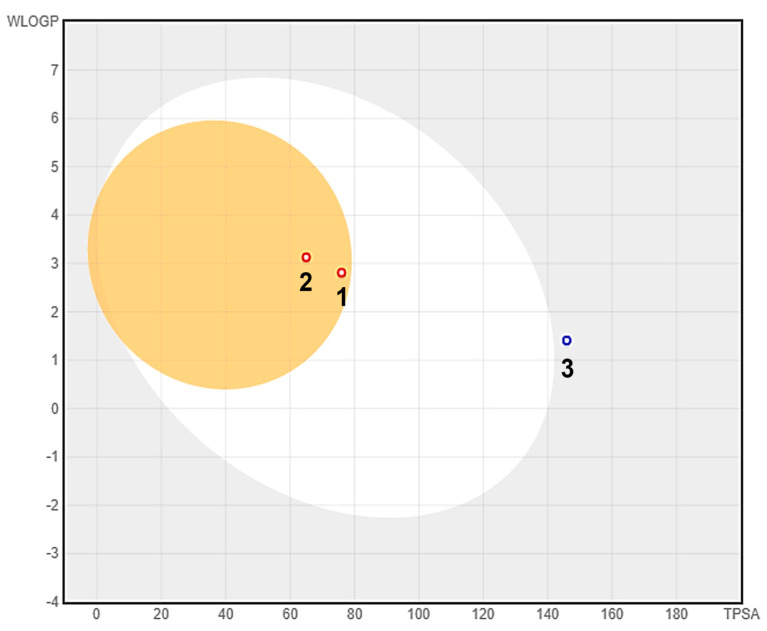
Prediction of GI tract and brain permeation of compounds **1**–**3** by brain or the intestinal estimated permeation predictive model (BOILED-Egg) method.

Compounds **1–3** were assessed for their adsorption, metabolism, distribution, and excretion (ADME) properties in silico to provide a prediction of their potential pharmacokinetic behavior (Table S1). Lipinski’s ‘rule of five’ (LRo5) was used to evaluate the pharmacokinetic profile of the compounds, which considers the following key physiochemical parameters: molecular weight (less than 500 Da), lipophilicity (MLogP less than 5), number of hydrogen bond donor (no more than 5), and number of hydrogen bond acceptors (no more than 10). As per LRo5, all three compounds exhibited good bioavailability and drug-likeness, with no violations against Lipinski parameters. Additionally, the compounds were subjected to a predictive brain or intestinal permeation modelling (BOILED-Egg) based on two functions, lipophilicity and total polar surface area [22]. From the BOILED-Egg plot (Figure 6), compounds **1** and **2**, located at the yellow region (yolk), are predicted to have a high propensity for blood–brain barrier (BBB) permeation. Compound **3**, on the hand, is predicted to be a non-BBB permeant, but is considered to have low gastrointestinal absorption, as it is slightly off the white region of the plot. Osiris Property Explorer was used to predict the toxicities of the compounds, specifically their potential mutagenicity, tumorigenicity, irritant effect, and reproductive toxicity (Table S2). All compounds were predicted to demonstrate mutagenic effects, which is due to the presence of a naphthalene fragment in their structures. However, structural manipulation, such as derivatization around the naphthalene core, may improve the compounds’ toxicity profiles.

## 3. Materials and Methods

### 3.1. General Experimental Procedures

Specific optical rotations ([α]_D_) were measured on a PerkinElmer 241 polarimeter in a in 2.0 mm × 100 mm cell at 20 °C. UV-vis spectra were obtained on a Shimadzu UV-2450 spectrophotometer with a 1 cm quartz cell. IR spectra were measured either on a PerkinElmer Spectrum Two FT-IR spectrophotometer or a Shimadzu Prestige-21 spectrophotometer coupled with Diffuse Reflectance Spectroscopy in KBr. Nuclear magnetic resonance (NMR) spectra were obtained either on a Varian VNMRS-500 MHz or a Agilent DD2 MR Varian- 500 MHz (^1^H 500 MHz, ^13^C 125 MHz). Spectra were acquired at 25 °C in MeOH-*d*_4_, with referencing to residual ^1^H or ^13^C signals in the deuterated solvent. HR-ESI mass spectra were measured on the use of the Agilent 6200 series TOF and 6500 series Q-TOF LC/MS system. The HPLC-DAD purification was carried out on a Shimadzu Prominence Liquid Chromatograph LC-20AT coupled with a SPD-M20A Photodiode Array Detector (Shimadzu Corp., Tokyo, Japan) and a semi-preparative reversed phase C_18_ column, Inertsil ODS-3 (10 mm I.D. × 250 mm, 5 μm, G.L. Sciences, Tokyo, Japan). The mobile phase was composed of ultrapure water (Milli-Q, Millipore, Schwalbach, Germany) as solvent A and acetonitrile (HPLC grade) as solvent B. Normal phase gravity column chromatography utilized silica gel 60 (Merck Art. 7734 and 9835). Aluminum TLC sheets pre-coated with silica gel 60 F254 (Merck Art. 1.05557) were used for routine analysis. TLC spots were visualized under UV light (254 nm and 365 nm) followed by spraying with a vanillin-sulfuric acid reagent.

### 3.2. Fungal Material and Fermentation

The voucher specimen is deposited at the Mae Fah Luang University Culture Collection, Chiang Rai, Thailand with designation numbers, MFLU 15-0030 and MFLUCC 15-1405 for ex-type culture and holotype specimen, respectively [13].

The fungus was cultivated on solid rice media (70 g brown rice, 0.3 g peptone, 0.1 g corn syrup, and 100 mL ultrapure water) in 15 × 1000 mL sterilized Fernbach culture flasks, followed by autoclaving (120 °C, 20 min). Five agar blocks of well-grown fungal culture in a malt extract agar plate were inoculated in the culture flasks and incubated under static conditions in a dark room at 25–30 °C, which lasted for 12 weeks until the fungal hyphae proliferated and the rice medium turned black in color.

### 3.3. Extraction and Isolation

The rice cultures were homogenized using a sterile metal spatula. Fermentation was terminated by the addition of EtOAc (3 × 300 mL) and the combined extracts were concentrated in a rotary evaporator to afford the crude extract (10.2 g). The crude EtOAc extract was reconstituted with 300 mL 10% aqueous MeOH and partitioned with *n*-heptane (3 × 100 mL). The combined organic layer was concentrated in vacuo to afford 2.73 g methanolic crude extract.

The resulting crude methanolic extract was fractionated, eluting initially with petroleum ether–EtOAc (1:1 and 2:3) followed by 20% increments of dichloromethane in methanol and finally methanol to afford 5 fractions. Fraction 1 (2.22 g) was chromatographed using petroleum ether–EtOAc (6:1, 3:1 and 1:1), EtOAc–MeOH (4:1 and 9:1) to yield 5 subfractions. Subfraction 1.5 was further purified twice using petroleum ether–EtOAc (1:12), EtOAc–MeOH (12:1, 7:3 and 1:1) followed by semi-preparative reversed phase HPLC by gradient elution using 40% solvent B for 5 min, 40%–100% solvent B for 20 min, 100% solvent B for 5 min and 100%–40% solvent B for 5 min to afford compound **1** (4.3 mg, *t*_R_ = 16.8 min). Compound **2** was obtained from the first subfraction of 1.5, which was purified by silica gel column chromatography using pure dichloromethane and dichloromethane–MeOH (13:1) followed by semi-preparative reversed phase HPLC using similar gradient conditions used for the purification of **1** to afford **2** (13.4 mg, *t*_R_ = 22.8 min). A subfraction 1.2 was column-chromatographed using a petroleum ether and a mixture of petroleum ether– CH_2_Cl_2_ (1:4, 1:8 and 1:9) and CH_2_Cl_2_–MeOH (9:1), followed by semi-preparative reversed phase HPLC employing similar gradient solvent system to afford compound **3** (3.5 mg, *t*_R_ = 23.1 min).

Sparticatechol A (**1**): brown syrup; [α]_D_^25^ +120 (*c* 0.1, MeOH); TLC (CH_2_Cl_2_:MeOH, 9:1 *v*/*v*): R_f_ = 0.17; UV (*c* 0.1, ACN) λ_max_ (log ε) 226 (5.0), 254 (4.7), 300 (4.5), 314 (4.4), 328 (4.4) nm; ^1^H NMR (CD_3_OD, 600 MHz), and ^13^C NMR (CD_3_OD, 600 MHz) see Table 1; IR (KBr): 3040, 1610, 1380, 1275, 1160, 1110, 1060, 955, 910, 870, 835, 755, 710 cm^−1^ HR-ESI-MS *m/z*: [M + H]^+^ calcd for C_23_H_23_O_9_, 443.1342; found, 443.1330; *m/z* [M + H – H_2_O]^+^ calcd for C_23_H_21_O_8_, 425.1236; found, 425.1226; *m/z* [M + Na]^+^ calcd for C_23_H_22_NaO_9_, 465.1162; found, 465.1152. IUPAC nomenclature: (8*S*)-(22*R*)-2,3-dihydroxypropyl-3-(2-(2,3-dihydroxyphenyl)naphtho[1,8-de][1,3]dioxin-2-yl)-3-hydroxypropanoate.

Sparticolin H (**2**): dark brown syrup; [α]_D_^25^ +104 (*c* 0.1, MeOH); TLC (CH_2_Cl_2_:MeOH, 9:1 *v*/*v*): R_f_ = 0.43; UV (*c* 0.1, ACN) λ_max_ (log ε) 226 (4.9), 300 (4.3), 314 (4.3), 328 (4.2) nm; ^1^H NMR (CD_3_OD, 500 MHz), and ^13^C NMR (CD_3_OD, 125 MHz) see Table 1; IR (KBr): 1712, 1636, 1607, 1584, 1411, 1380, 1273, 1143, 1073, 1037, 957, 895, 820, 794, 756, 666 cm^−1^; HR-ESI-MS *m/z*: [M + H]^+^ calcd for C_19_H_17_O_5_, 325.1071; found, 325.1066; *m/z* [M + Na]^+^ calcd for C_19_H_16_NaO_5_, 347.0890; found, 347.0889. IUPAC nomenclature: 2-((2*R*,3a*R*,6a*S*)-2,3,3a,6a-tetrahydrospiro[cyclopenta[b]furan-4,2’-naphtho[1,8-de][1,3]dioxin]-2-yl)acetic acid.

### 3.4. Biological Assays

Anti-cyclooxygenase (COX) Assay. The cyclooxygenase inhibitory potential of compounds **1–3** were assessed using the COX Activity Assay Kit (Catalog No. 760151, Cayman Chemical, Ann Arbor, MI, USA). The assay kit quantifies the peroxidase activity of cyclooxygenases by observing colorimetrically the oxidation of N,N,N′,N′-tetramethyl-p-phenylenediamine (TMPD) at 590 nm. The samples were dissolved in DMSO with concentrations ranging from 0.1 to 10 µg/mL. Celecoxib was used as a reference compound. The assay was carried out according to the protocol provided by the manufacturer and the half-maximal inhibitory concentration of the compounds were determined using concentration-response curve in triplicate measurements.

Anti-proliferation and Cytotoxicity Assay. Compounds **1**–**3** were assayed against human umbilical vein endothelial cells (HUVEC, ATCC CRL-1730) and K562 human chronic myeloid leukemia cells (DSM ACC 10) for their antiproliferative effects (GI_50_). Cytotoxicity properties were also assessed against several mammalian cancer cell lines including mouse fibroblast L929 (ACC 2), HeLa (KB3.1), human breast adenocarcinoma (MCF-7, ACC 115), adenocarcinomic human alveolar basal epithelial cells (A549, ACC 107), human prostate cancer cells (PC-3, ACC 465), ovarian carcinoma (SKOV-3, HTB-77) and squamous cell carcinoma (A431, ACC 91), which was expressed as IC_50_ by MTT Assay [23] and against HeLa human cervix carcinoma cells (DSM ACC 57), expressed as CC_50_ by Cell Titer Blue Assay [24]. Inhibitory concentrations are provided as 50% half-maximal inhibitory concentration (IC_50_, concentration of the substance where a specific biological process is reduced by half), 50% inhibition of cell growth (GI_50_, the concentration needed to reduce the growth of treated cells to half that of untreated cells) or 50% cytotoxic concentration (CC_50_, the concentration that kills 50% of treated cells).

Antimicrobial Assay. The antimicrobial activities of **1**–**3** were evaluated against various fungal and bacterial strains. The minimum inhibitory concentration (MIC) of the tested compounds were determined in a sterile 96-well plates by the broth microdilution method according to our previously described procedures [25]. Gentamicin (MIC vs. *P. aeruginosa* = 0.21 µg/mL), kanamycin (MIC vs. *M. smegmatis* = 1.7 µg/mL), nystatin (MIC vs*. R. glutinis* = 2.1 µg/mL; MIC vs. *C. albicans*, *P. anomala*, *M. hiemalis*, *S. pombe* = 4.2 µg/mL), ciprobay (MIC vs. *A. baumannii* = 0.26 µg/mL) and oxytetracycline hydrochloride (MIC vs. *S. aureus* = 0.21 µg/mL; MIC vs. *C. violaceum* = 0.83 µg/mL; MIC vs. *E. coli* = 3.3 µg/mL; MIC vs. *B. subtilis* = 8.3 µg/mL). Nystatin was used as a positive control against fungi while ciprobay, gentamicin, kanamycin and oxytetracyclin were used as a positive control against Gram-positive and Gram-negative bacteria, respectively.

Antituberculosis Assay. Antituberculosis inhibitory activity of the compounds against replicating Mycobacterium tuberculosis H_37_Rv (American Type Culture Collection, Rockville, MD, USA) was determined using a fluorescence reading at 530 nm excitation wavelength and 590 nm emission wavelength in the Microplate Alamar Blue Assay (MABA) [26]. The MIC values was determined as the lowest concentration exhibiting a 90% fluorescence inhibition compared to the untreated bacterial control. Rifampin (MIC = 0.15 μg/mL), isoniazid (MIC = 0.63 μg/mL), and streptomycin (MIC = 0.83 μg/mL) were used as positive drug controls.

### 3.5. Computational Calculations

Theoretical ECD Calculations. A conformational analysis on both sparticatechol A (**1**) and sparticol H (**2**) was performed using the Avogadro (version 1.1.1) platform, which included a search for low-energy conformations using the MMFF94 molecular mechanics force field and conformer optimization following the steepest descent algorithm. The geometries of all stable conformers were re-optimized via density functional theory calculations at a B3LYP/6-31G(d) basis set using acetonitrile as a solvent model on a polarizable continuum model (PCM). Boltzmann population distribution was estimated for each conformer based on the calculated energies, taken as the sum of electronic and zero-point energies. The optimized geometries were subjected time-dependent DFT (TDDFT) using B3LYP/6-31G(d), WB97XD/DGDZVP, and B97D/TZVP basis sets at gas phase and acetonitrile PCM solvent model. A Gaussian distribution function was used to generate the ECD curve from the calculated rotatory strength values with 3000 cm^−1^ half-height width. All DFT calculations were carried out using Gaussian 16W while the visualization of results was done on GaussView 6.0 [27].

Molecular docking studies. Compound **1** was subjected to molecular docking analysis against COX-2 (PDB ID: 3LN1). All molecular docking experiments were performed on UCSF Chimera platform (version 1.14.1) (University of California-San Francisco, CA, United States) [28]. The three-dimensional structure of COX-2 was added to the docking platform as PDB format. The receptor was prepared by Each protein crystal structure removing all co-crystallized ligands and water molecules. Meanwhile, compounds **1**–**3** as ligands, were added to the docking platform as the SYBYL mol2 file, which were pre-optimized using the using the MMFF94 force field via the steepest descent algorithm using Avogadro software. Minimization and dock-prepping of ligand and protein structures were done using Antechamber [29], and molecular docking was performed using the BFGS algorithm of AutoDock Vina (version 1.1.2) [30]. The conformational protein–ligand structure was visualized and analyzed using Biovia Discovery Studios (version 4.1).

ADMET Profiling. In silico prediction of the adsorption, distribution, metabolism, and excretion (ADME) properties of compounds **1**–**3** was carried out using SwissADME software (Molecular Modeling Group, Swiss Institute of Bioinformatics, Lausanne, Switzerland, 2019) [22,31]. Pharmacokinetic profiles of the compounds were assessed based on the Lipinski’s ‘rule of five’, which predicts a compound’s oral druggability. Additionally, OSIRIS property explorer program (Thomas Sander, Idorsia Pharmaceuticals Ltd., Allschwil, Switzerland, 2017) was employed for the in silico prediction of toxicity prediction, particularly mutagenicity, tumorigenicity, irritant effects, and reproductive toxicity of the metabolites [19].

## 4. Conclusions

This study investigated the biologically active chemical constituents of the rice culture of *Sparticola junci*, where two highly oxygenated naphthoketal-bearing polyketides, sparticatechol A (**1**), sparticolin H (**2**), along with sparticolin A (**3**), were isolated and identified. Compounds **1** and **2** inhibited the two cyclooxygenase isozymes, COX-1 and COX-2 illustrating its anti-inflammatory potentials. In silico molecular docking analysis of **1** showed preferential binding in the NSAID active site. In addition, **1** and **2** also demonstrated weak antiproliferative effects against myeloid leukemia K-562 cell lines and cytotoxic activity against HeLa cervical carcinoma cell lines. Compounds **1**–**3** exhibited good bioavailability and drug-likeness, with no violations against Lipinski parameters. Our findings in general establish the potential of naphthoketal derivatives **1**–**3** as a possible drug inspiration for discovering new anti-inflammatory and cancer agents.

## Figures and Tables

**Table 1:** NMR Spectroscopic Data of **1** and **2** in MeOH-*d*_4_.

Position	1		2	
	*δ_H_*^a^ (Mult., *J* in Hz)	*δ_C_*^a^, Type	*δ_H_*^b^ (Mult., *J* in Hz)	*δ_C_*^c^, Type
1	-	105.1, C	-	111.0, C
2	-	123.5, C	5.88 (ddd, 5.7, 1.0, 0.4)	130.1, CH
3	-	145.1, C	6.25 (dd, 5.7, 1.7)	139.3, CH
4	-	147.3, C	5.07 (ddd, 7.0, 1.8, 1.0)	85.7, CH
5	6.59 (dd, 7.9, 1.5)	117.0, CH	3.14 (td, 8.5, 7.1)	51.1, CH
6	6.42 (dd, 7.9, 1.5)	119.8, CH	1.86–1.73 (m)	32.7, CH_2_
7	6.80 (dd, 7.9, 1.5)	121.6, CH	4.24 (dtd, 12.1, 6.1, 5.6)	78.7, CH
8	4.81 (dd, 14.0, 3.1)	73.9, CH	2.52 (d, 6.3)	39.9, CH_2_
9a	2.68 (dd, 15.7, 9.4)	38.0, CH_2_	-	173.5, C
9b	2.97 (dd, 15.7, 3.2)			
10	-	173.1, C	-	148.2, C
11	-	148.7, C	6.87 (dd, 8.9, 7.4)	108.6, CH
12	7.02 (dd, 8.3, 1.3)	110.2, CH	7.40 (dd, 8.9, 7.4)	127.1, CH
13	7.37 (dd, 8.3)	121.6, CH	7.47 (dd, 8.9, 7.4)	120.1, CH
14	7.39 (dd, 8.3, 1.3)	128.2, CH	-	134.5, C
15	-	135.5, C	7.47 (dd, 8.9, 7.4)	120.1, CH
16	7.39 (dd, 8.3, 1.3)	128.2, CH	7.40 (dd, 8.9, 7.4)	127.1, CH
17	7.37 (dd, 8.3)	121.6, CH	6.87 (dd, 8.9, 7.4)	108.6, CH
18	7.02 (dd, 8.3, 1.3)	110.2, CH	-	148.4, C
19	-	148.9, C	-	114.1, C
20	-	115.5, C	-	-
21a	4.10 (td, 11.4, 6.3)	66.9, CH_2_	-	-
21b	4.19 (td, 11.4, 4.3)		-	-
22	3.80 (dt, 15.2, 6.7)	71.1, CH	-	-
23	3.56 (ddd, 7.3, 5.7, 2.3)	64.0, CH_2_	-	-

^a^ Recorded at 600 MHz, ^b^ Recorded at 500 MHz, ^c^ Recorded at 125 MHz; Carbon multiplicities were deduced from HSQC-DEPT-135 spectra. *δ_H_*: proton chemical shift; *δ_C_*: carbon chemical shift.

**Table 2:** Antiproliferative and cytotoxicity of compounds **1–3** against mammalian cancer cell lines.

Compound	Antiproliferative Effect GI_50_ (µM)		Cytotoxicity CC_50_ (µM)	Cytotoxicity IC_50_ (µM)	
	HUVEC	K-562	HeLa	L929	KB3.1
**1**	>50	97.5	85.3	−	−
**2**	>50	80.8	124.7	22.9	21.8
**3**	>50	91.3	119.8	−	−
Imatinib	18.5	0.17	65.8	N.D.	N.D.
Epothilone B	N.D.	N.D.	N.D.	1.57 × 10^−3^	3.9 × 10^−3^

N.D.: Not determined; Em dash (−): no observed cytotoxic activity.

**Table 3:** In vitro Cyclooxygenase inhibitory activity of compounds **1**–**3**.

Compound	COX-1 IC_50_ (µM)	COX-2 IC_50_ (µM)
**1**	8.8 × 10^−3^	0.3
**2**	1.8	1.5
**3**	1.4	2.3
Celecoxib	18.09	0.656

IC_50_ values represented as mean based on triplicate measurements.

## Data Availability

NMR and HRMS spectroscopic data are available at the Research Center for the Natural and Applied Sciences, University of Santo Tomas, Philippines and Institute of Organic Chemistry, University of Regensburg, Germany (AR), while the MIC vs pathogens and IC_50_ or GI_50_ or CC_50_ vs cancer cell-lines data are available at the Helmholtz Center for Infection Research, Germany and Leibniz Institute of Natural Products, Germany. IC_50_ and binding energy values vs COX enzymes are available at the Research Center for the Natural and Applied Sciences, University of Santo Tomas, Philippines.

## Supplementary Materials

The following are available online at https://www.mdpi.com/article/10.3390/ijms222212379/s1.
