# Supplementary material for: COX Inhibitory and Cytotoxic Naphthoketal-Bearing Polyketides from Sparticola junci
Source: Int J Mol Sci. 2021 Nov 17;22(22):12379. doi: 10.3390/ijms222212379 (PMC8619024; doi:10.3390/ijms222212379)
Supplement: Supplementary file 1 [file ijms-22-12379-s001.zip › ijms-1444710-supplementary.pdf]

SUPPLEMENTARY INFORMATION

COX Inhibitory and Cytotoxic Naphthoketal-Bearing Polyketides from *Sparticola junci*

Katherine Yasmin M. Garcia[1,2], Mark Tristan J. Quimque[1,2,3], Gian Primahana[4,5,6], Andreas Ratzenböck[7], Mark Joseph B. Cano[1,2], Jeremiah Francis A. Llaguno[2], Hans-Martin Dahse[8], Chayanard Phukhamsakda[9,10], Frank Surup[4,5], Marc Stadler[4,5] and Allan Patrick G. Macabeo [2,*]

[1] The Graduate School, University of Santo Tomas, España Blvd., Manila 1015, Philippines; katherineyasmin.garcia.gs@ust.edu.ph (K.Y.M.G.); marktristan.quimque@g.msuiit.edu.ph (M.T.J.Q.); markjoseph.cano.gs@ust.edu.ph (M.J.B.C.)

[2] Laboratory for Organic Reactivity, Discovery and Synthesis (LORDS), Research Center for the Natural and Applied Sciences, University of Santo Tomas, España Blvd., Manila 1015, Philippines; jeremiahfrancis.llaguno.sci@ust.edu.ph

[3] Chemistry Department, College of Science and Mathematics, Mindanao State University Iligan Institute of Technology, Tibanga, Iligan City 9200, Philippines

[4] Department of Microbial Drugs, Helmholtz Centre for Infection Research and German Centre for Infection Research (DZIF), Partner Site Hannover/Braunschweig, Inhoffenstrasse 7, 38124 Braunschweig, Germany; gian.primahana@helmholtz-hzi.de (G.P.); frank.surup@helmholtz-hzi.de (F.S.); marc.stadler@helmholtz-hzi.de (M.S.)

[5] Institute of Microbiology, Technische Universität Braunschweig, Spielmannstraße 7, 38106 Braunschweig, Germany

[6] Research Center for Chemistry, National Research and Innovation Agency (BRIN), Kawasan Puspitek, Serpong, Tangerang Selatan 15314, Indonesia

[7] Institut für Organische Chemie, Universität Regensburg, Universitätstrasse 31, 93053 Regensburg, Germany; andreas.ratzenboeck@chemie.uni-regensburg.de

[8] Leibniz-Institute for Natural Product Research and Infection Biology, Hans-Knöll-Institute (HKI), 07745 Jena, Germany; hans-martin.dahse@hki-jena.de

[9] Center of Excellence in Fungal Research, Mae Fah Luang University, Chiang Rai 57100, Thailand; chayanard91@gmail.com

[10] Institute of Plant Protection, College of Agriculture, Engineering Research Center of Chinese Ministry of Education for Edible and Medicinal Fungi, Jilin Agricultural University, Changchun 130118, China

*Correspondence: agmacabeo@ust.edu.ph

LIST OF SUPPORTING INFORMATION

	Page
**Figure S1.** ^{1}H NMR spectrum (MeOH-d_4, 600 MHz) of sparticatechol A (**1**)	3
**Figure S2.** ^{13}C NMR spectrum (MeOH-d_4, 600 MHz) of sparticatechol A (**1**)	3
**Figure S3.** HSQC-DEPT spectrum of sparticatechol A (**1**)	4
**Figure S4.** COSY spectrum of sparticatechol A (**1**)	4
**Figure S5.** HMBC spectrum of sparticatechol A (**1**)	5
**Figure S6.** ROESY spectrum of sparticatechol A (**1**)	5
**Figure S7.** HR-ESIMS spectrum of sparticatechol A (**1**)	6
**Figure S8.** ^{1}H NMR spectrum (MeOH-d_4, 500 MHz) of sparticolin H (**2**)	7
**Figure S9.** ^{13}C NMR spectrum (MeOH-d_4, 125 MHz) of sparticolin H (**2**)	7
**Figure S10.** HSQC-DEPT spectrum of sparticolin H (**2**)	8
**Figure S11.** COSY spectrum of sparticolin H (**2**)	8
**Figure S12.** HMBC spectrum of sparticolin H (**2**)	9
**Figure S13.** NOESY spectrum of sparticolin H (**2**)	9
**Figure S14.** HR-ESIMS spectrum of sparticolin H (**2**)	10
**Figure S15.** Low energy conformers (> 1%) of (8*S*,22*R*)-**1** optimized at B3LYP/6-31G(d) (PCM/MeOH).	11
**Table S1.** Lipinski's rule of five for ADME analysis of compounds **1-3**	12
**Table S2.** Predicted toxicity parameters of compounds **1-3**	12

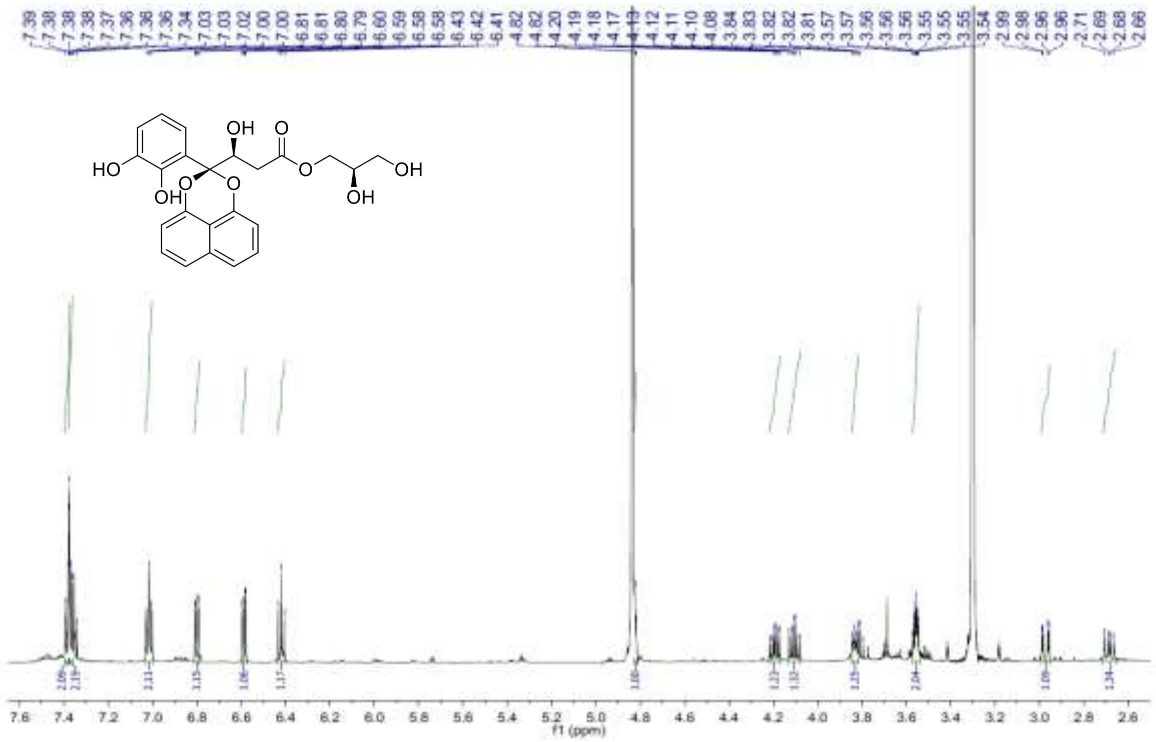

Figure S1. ^{1}H NMR spectrum (MeOH-d_4, 600 MHz) of sparticatechol A (**1**)

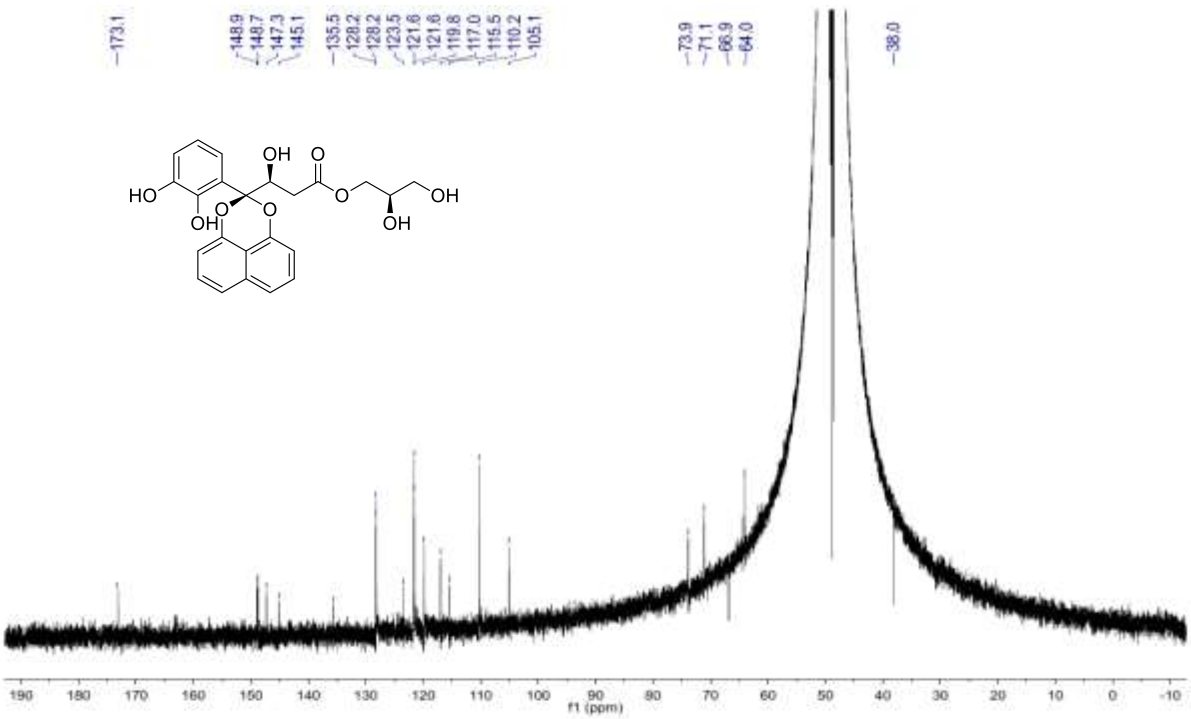

Figure S2. ^{13}C NMR spectrum (MeOH-d_4, 600 MHz) of sparticatechol A (**1**)

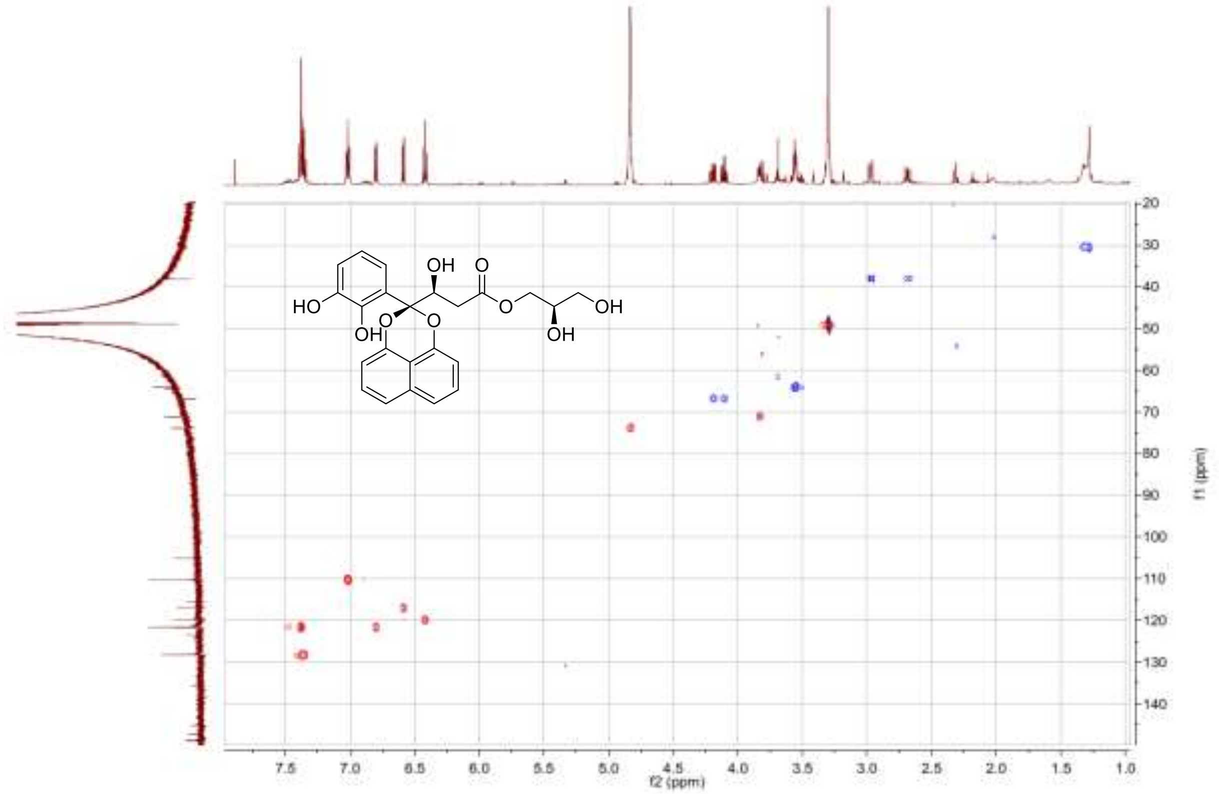

Figure S3. HSQC-DEPT spectrum of sparticatechol A (**1**)

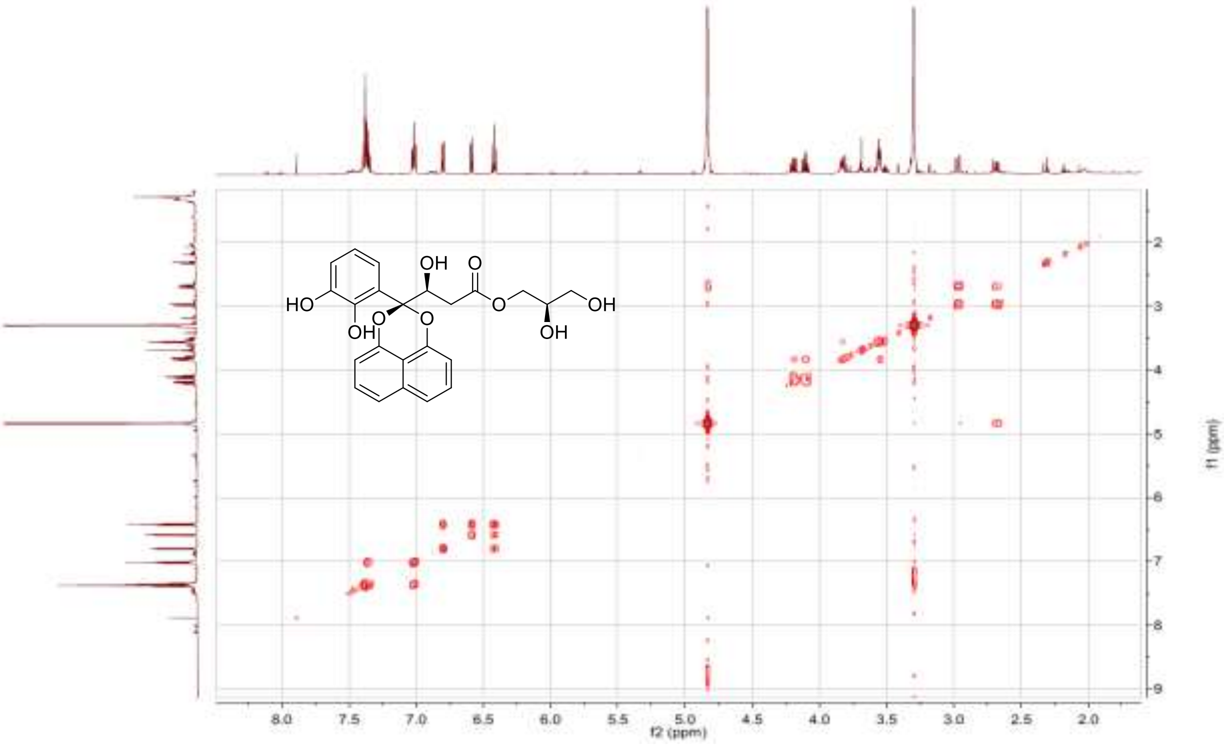

Figure S4. COSY spectrum of sparticatechol A (**1**)

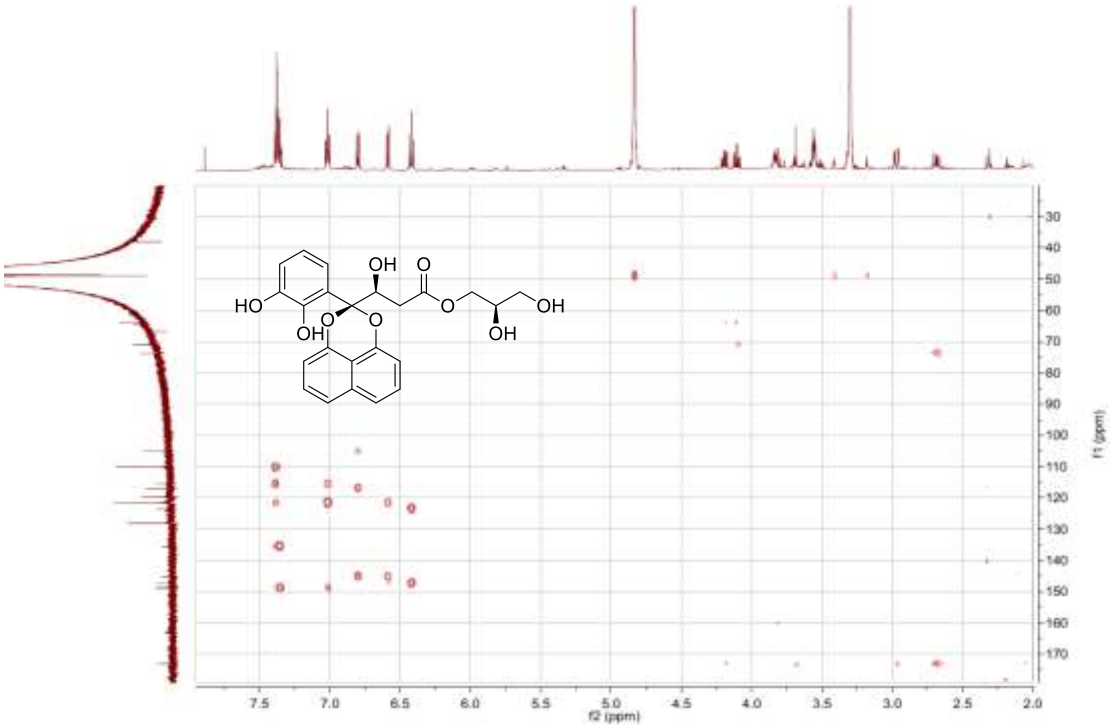

Figure S5. HMBC spectrum of sparticatechol A (**1**)

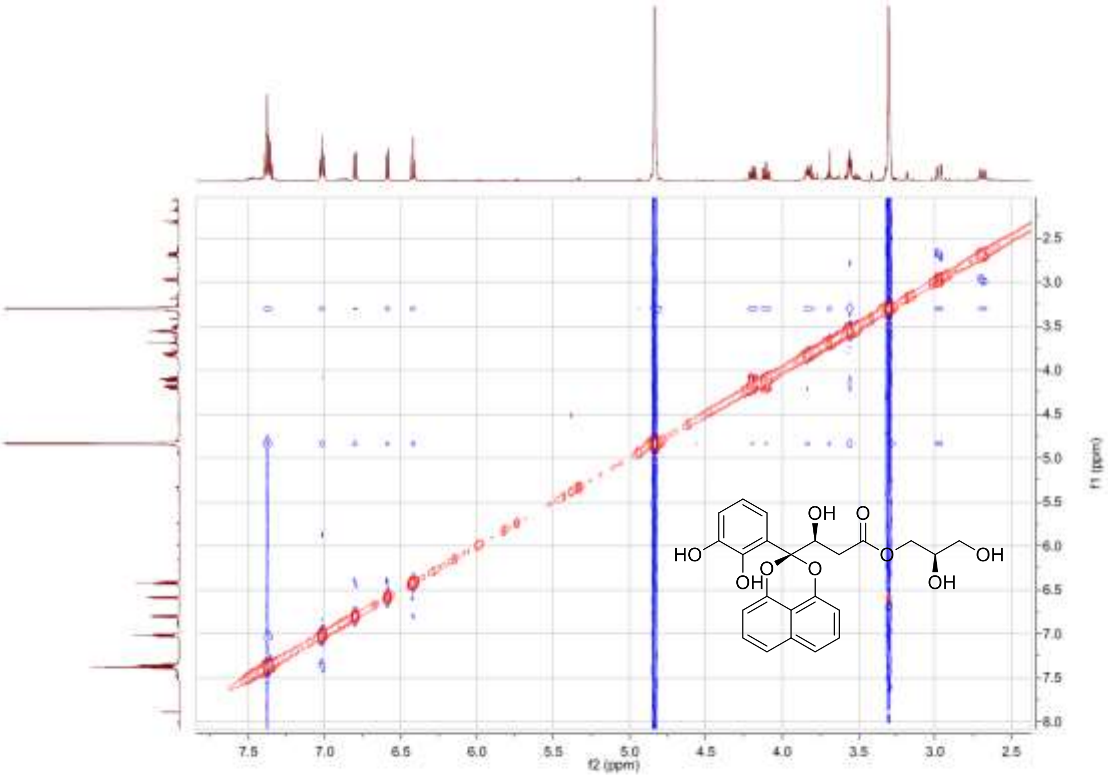

Figure S6. ROESY spectrum of sparticatechol A (**1**)

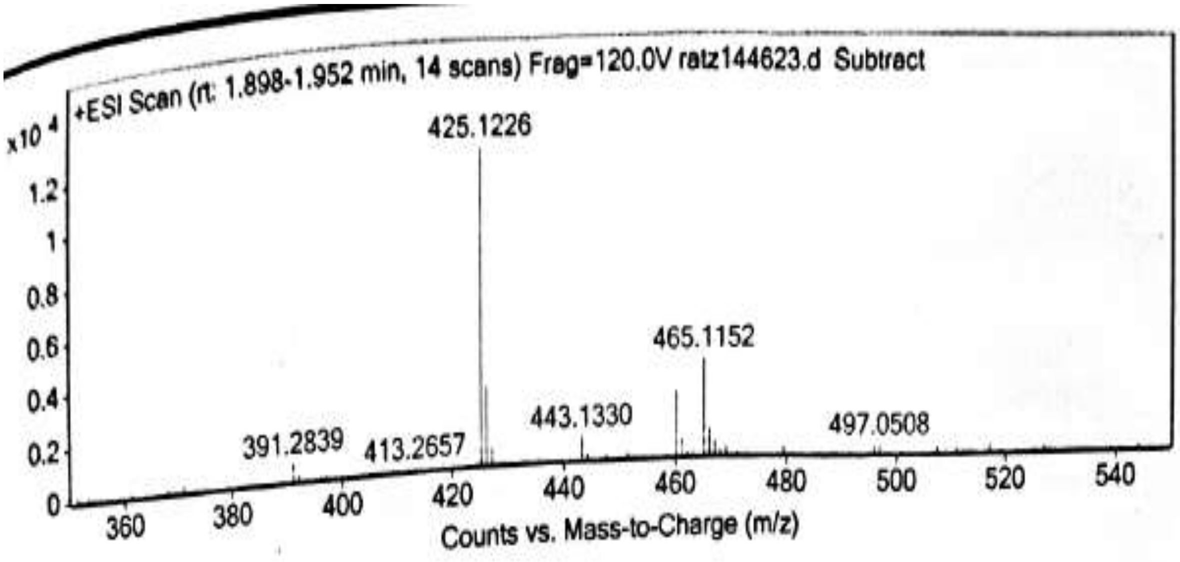

Figure S7. HR-ESIMS spectrum of sparticatechol A (**1**)

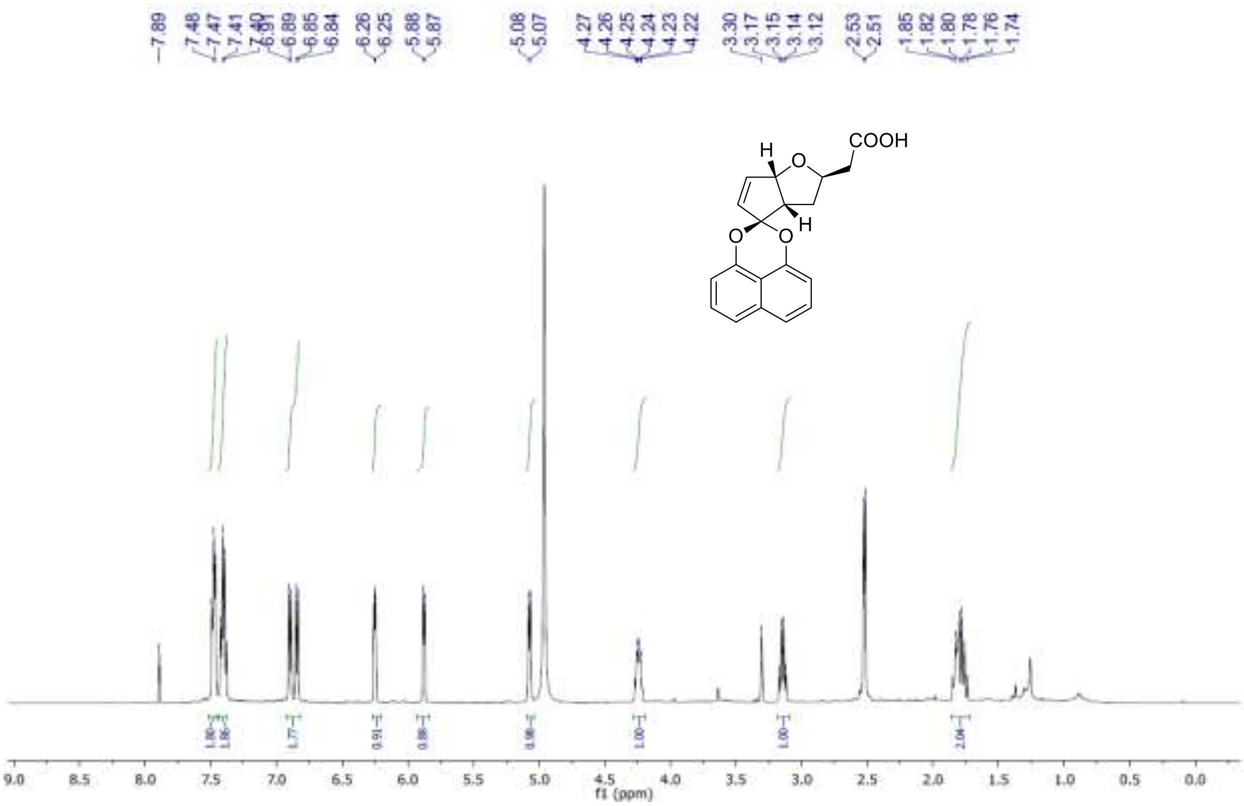

Figure S8. ^{1}H NMR spectrum (MeOH-d_4, 500 MHz) of sparticolin H (**2**)

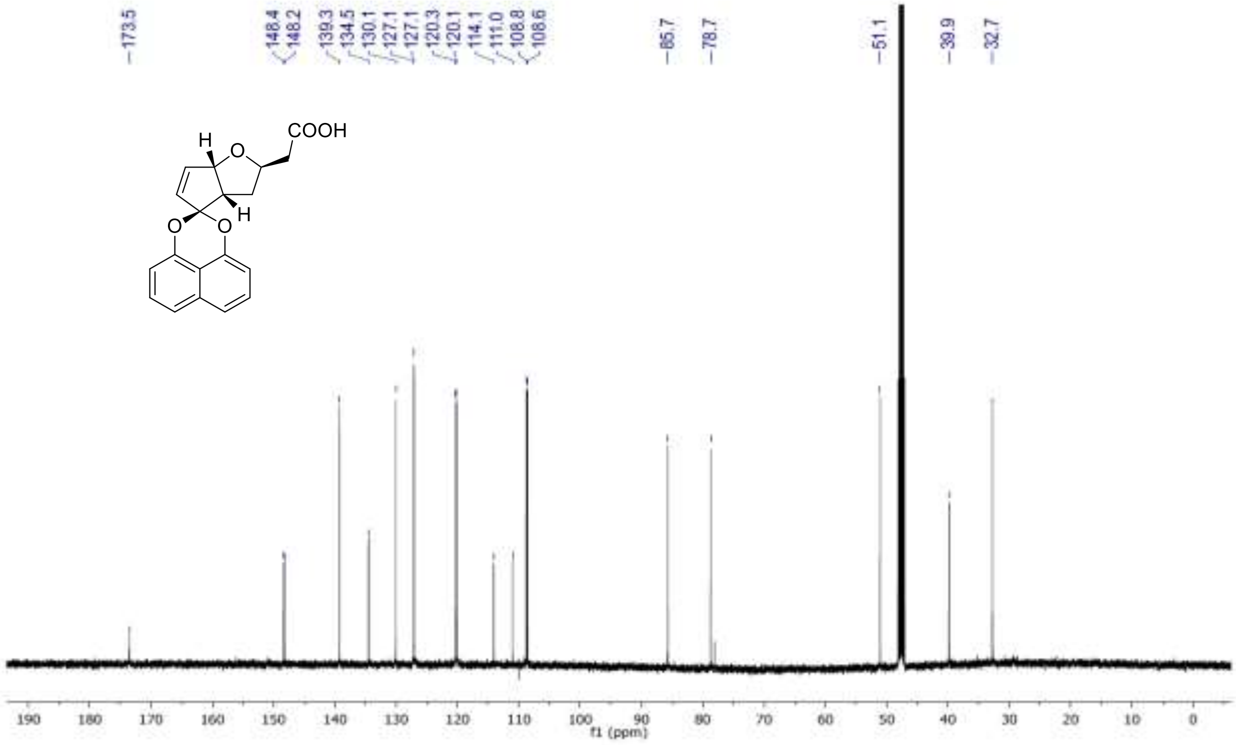

Figure S9. ^{13}C NMR spectrum (MeOH-d_4, 125 MHz) of sparticolin H (**2**)

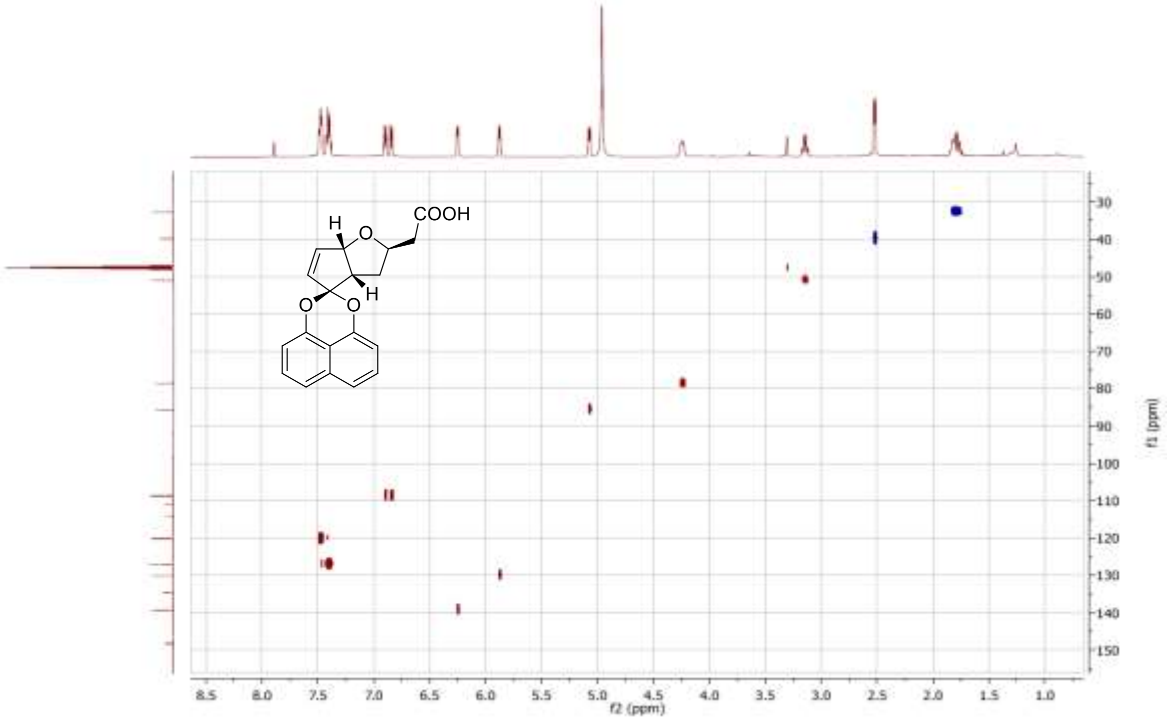

Figure S10. HSQC spectrum of sparticolin H (**2**)

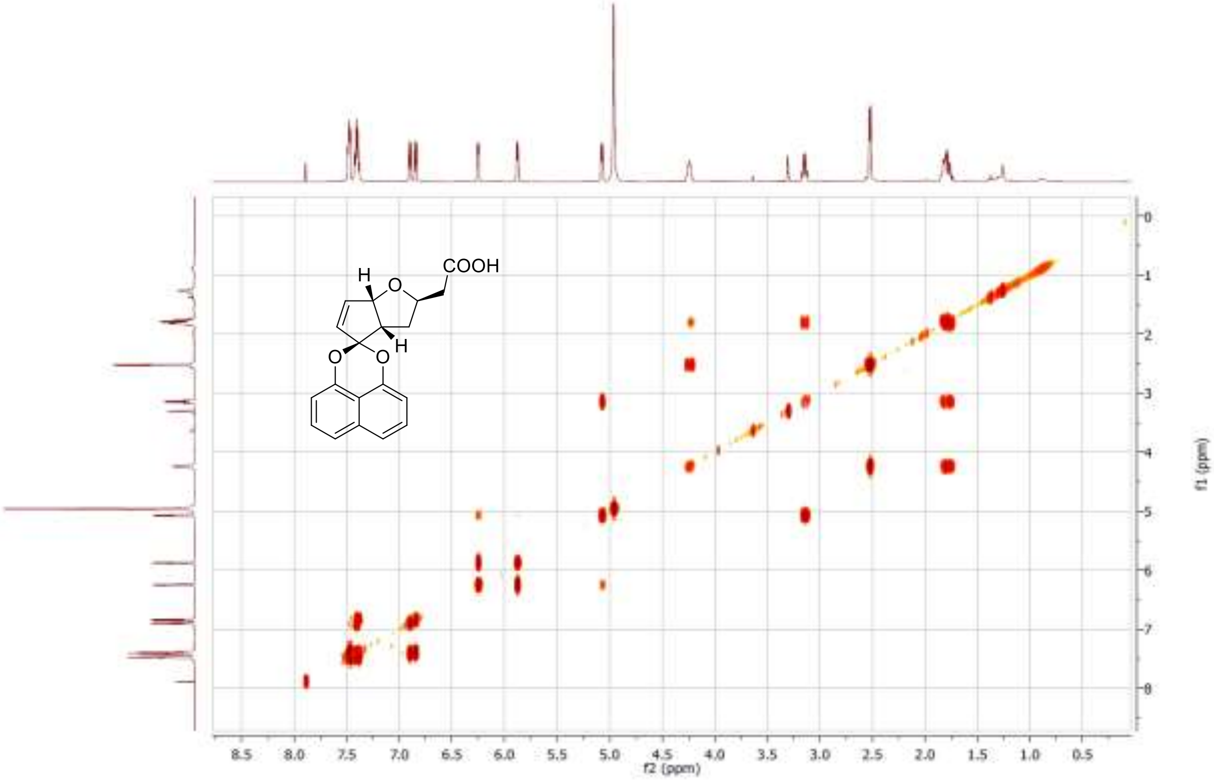

Figure S11. COSY spectrum of sparticolin H (**2**)

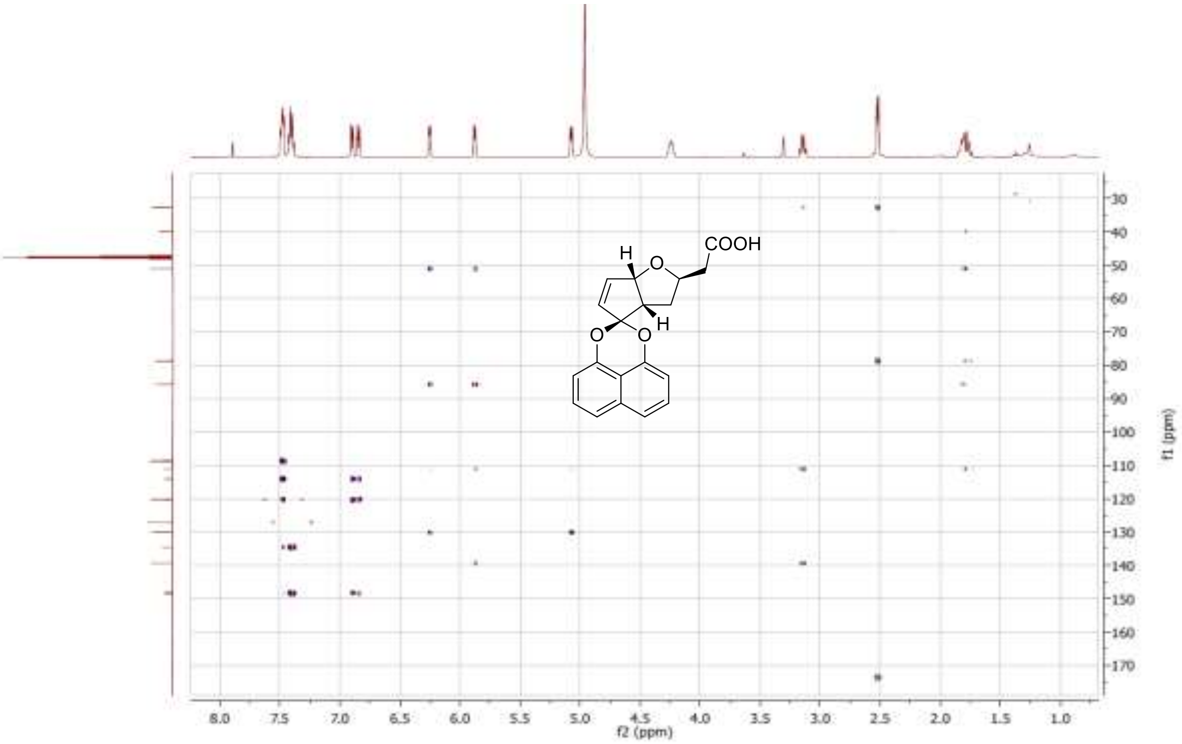

Figure S12. HMBC spectrum of sparticolin H (**2**)

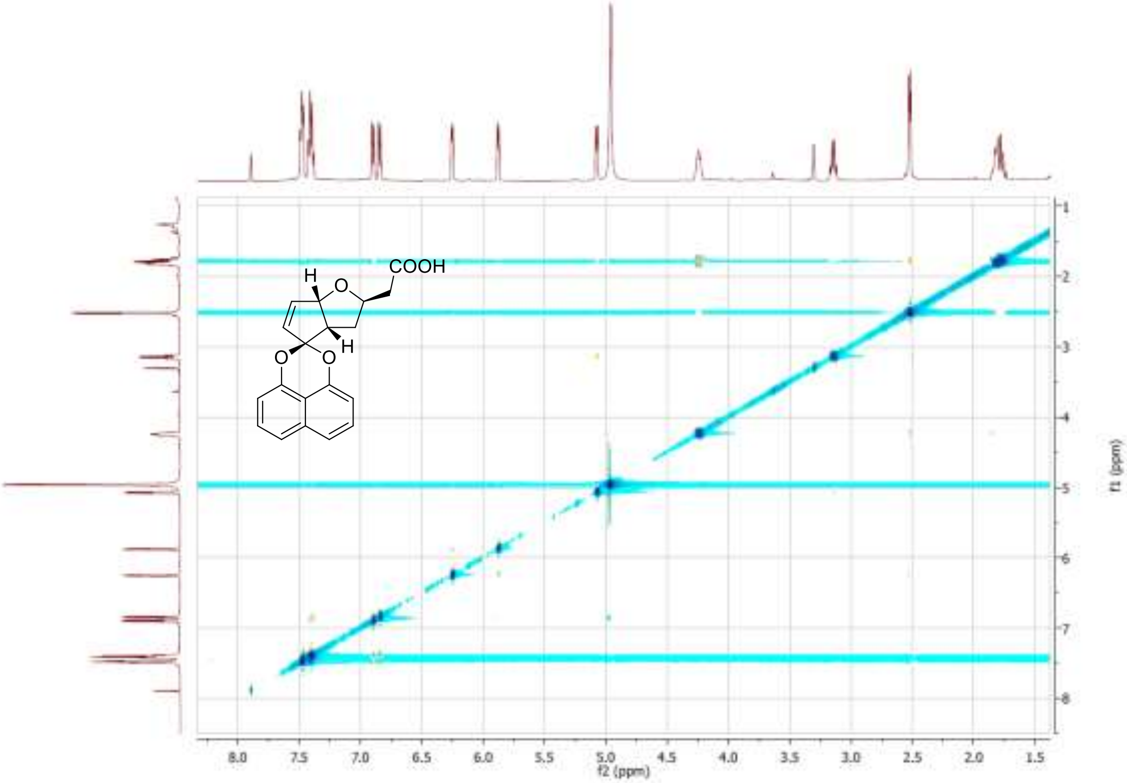

Figure S13. NOESY spectrum of sparticolin H (**2**)

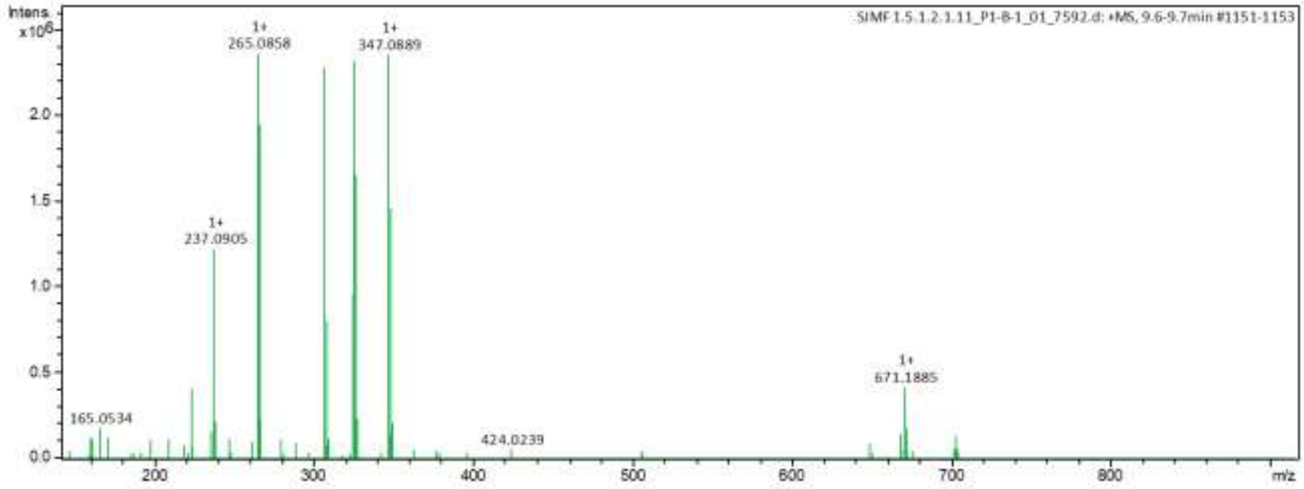

Figure S14. HR-ESIMS spectrum of sparticolin H (**2**)

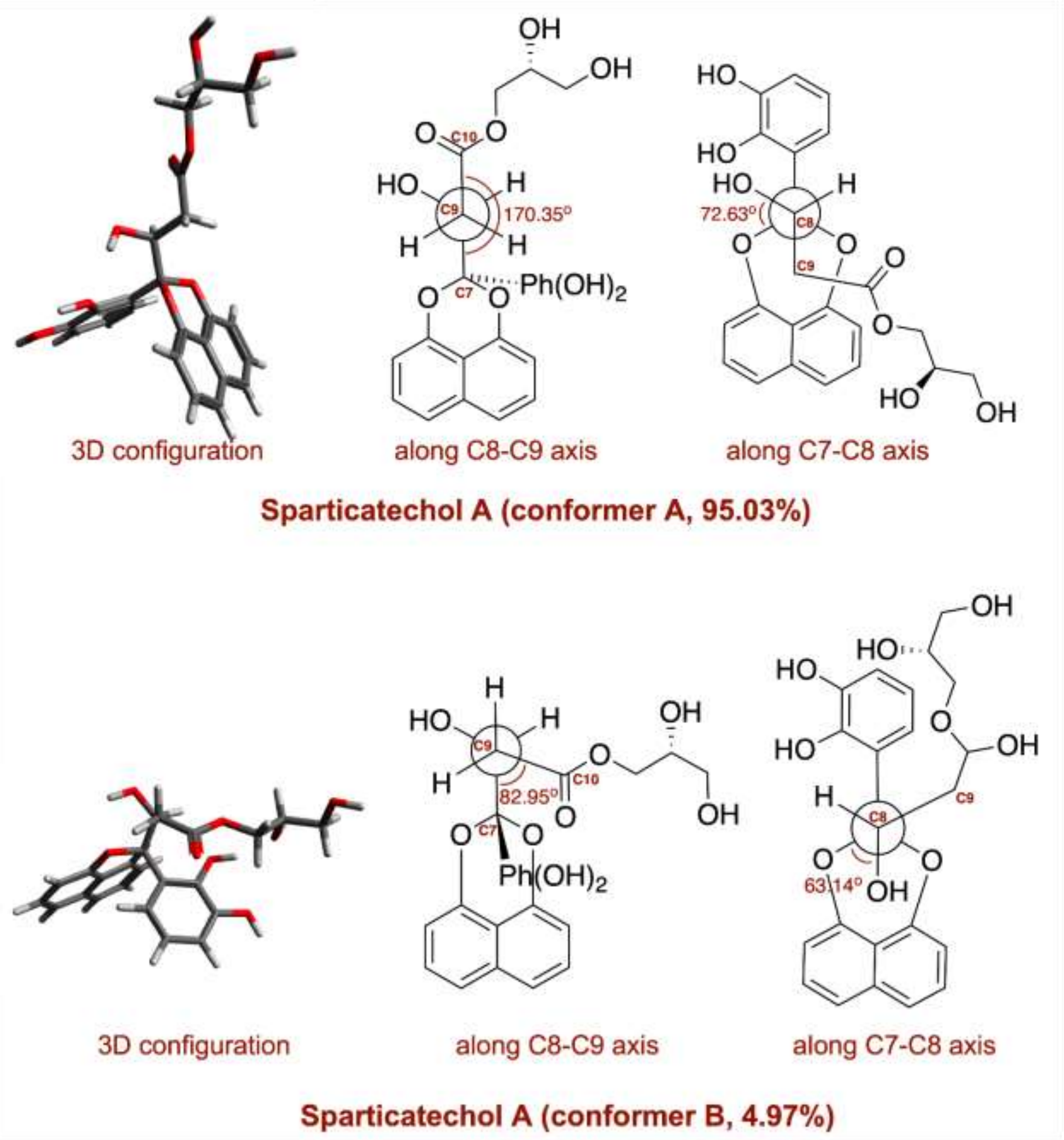

Figure S15. Low energy conformers (> 1%) of (8*S*,22*R*)-**1** optimized at B3LYP/6-31G(d) (PCM/MeOH).

Table S1. Lipinski's rule of five for ADME analysis of compounds **1**-**3**.

	**Lipinski's Rule of Five**					
Compound	Molecular weight (g/mol)	Lipophilicity (MLogP)	H-bond donor	H-bond acceptor	Rule violations	Drug-likeness
	< 500	< 5	≤ 5	≤ 10	<2	
**1**	442.42	0.67	5	9	0	yes
**2**	324.33	2.31	1	5	0	yes
**3**	322.31	2.16	2	5	0	yes

Table S2. Predicted toxicity parameters of compounds **1**-**3**.

**Compound**	**Mutagenicity**	**Tumorigenicity**	**Irritant Effect**	**Reproductive Toxicity**
**1**	High Risk	None	None	None
**2**	High Risk	None	None	None
**3**	High Risk	None	None	None
